# Connexins in Cancer: Jekyll or Hyde?

**DOI:** 10.3390/biom10121654

**Published:** 2020-12-10

**Authors:** Erin E. Mulkearns-Hubert, Ofer Reizes, Justin D. Lathia

**Affiliations:** 1 Department of Cardiovascular and Metabolic Sciences, Lerner Research Institute, Cleveland Clinic, Cleveland, OH 44195, USA; reizeso@ccf.org (O.R.); lathiaj@ccf.org (J.D.L.); 2 Case Comprehensive Cancer Center, Case Western Reserve University, Cleveland, OH 44106, USA; 3 Department of Molecular Medicine, Cleveland Clinic Lerner College, Medicine of Case Western Reserve University, Cleveland, OH 44195, USA; 4 Rose Ella Burkhardt Brain Tumor and Neuro-Oncology Center, Cleveland Clinic, Cleveland, OH, 44195, USA

**Keywords:** connexins, cancer, gap junctions, cancer stem cells, hemichannels

## Abstract

The expression, localization, and function of connexins, the protein subunits that comprise gap junctions, are often altered in cancer. In addition to cell–cell coupling through gap junction channels, connexins also form hemichannels that allow communication between the cell and the extracellular space and perform non-junctional intracellular activities. Historically, connexins have been considered tumor suppressors; however, they can also serve tumor-promoting functions in some contexts. Here, we review the literature surrounding connexins in cancer cells in terms of specific connexin functions and propose that connexins function upstream of most, if not all, of the hallmarks of cancer. The development of advanced connexin targeting approaches remains an opportunity for the field to further interrogate the role of connexins in cancer phenotypes, particularly through the use of in vivo models. More specific modulators of connexin function will both help elucidate the functions of connexins in cancer and advance connexin-specific therapies in the clinic.

## 1. Introduction

Communication between cells is essential for normal tissues to maintain the ability to grow and respond to their environment. However, this process is frequently altered in cancer cells. Over 50 years ago, Loewenstein and Kanno observed that liver cancer cells displayed a lack of cell–cell communication [1], and further studies supported this observation in other tumor types. This led to the long-standing historical dogma that connexins, the proteins that make up gap junctions (GJs), are functionally tumor suppressive. Over time, additional evidence has suggested a more complex system where connexins serve multiple cellular functions and individual connexins can act as both tumor promoters and tumor suppressors depending on context. In this review, we discuss the mechanisms—insofar as they are known—by which the connexin family of GJ proteins mediates the key phenotypes of cancer as laid out by Hanahan and Weinberg [2], including roles in more recently appreciated cancer phenotypes such as immune evasion and metabolic reprogramming.

### 1.1. Canonical and Non-Canonical Functions of Connexins

#### 1.1.1. Gap Junctions

Connexins are tetraspanin transmembrane proteins that assemble into a circular hexameric structure, termed a connexon, arranged around a central pore. Each connexin subunit contains two extracellular loops, which mediate docking between connexons on adjacent cells, and three intracellular regions: an intracellular loop and N- and C-terminal tails. When docked, the pore of the GJ allows molecules such as adenosine triphosphate (ATP) and other nucleotides, amino acids, small metabolites (including glucose), miRNAs (including miR-142, miR-223, miR-34a, and miR-124-3p [3,4,5]), second messengers (including cyclic adenosine monophosphate (cAMP) and inositol trisphosphate (IP_3_)), reactive oxygen species (ROS), glutathione, ions (Ca^2+^ and K^+^), and small proteins less than approximately 1.5 kDa to pass from the cytoplasm of one cell to another. Importantly, this transfer of materials is driven by simple diffusion gradients and is not an active transport. The opening and closing of GJ channels are mediated by multiple factors, including cross-channel pH and voltage, connexin phosphorylation, and intracellular Ca^2+^ concentration. There is evidence that channels composed of different connexin proteins display some varying selectivity to molecules, although the challenges associated with understanding exactly which molecules pass through GJs in a specific situation have limited a full understanding of channel selectivity. Furthermore, there is an emerging recognition that, in addition to their function in communication, GJ structures can function as adhesive anchors between cells (see [6]), particularly during cell motility, as well as protein scaffolds, as detailed in the section on non-junctional roles for connexins.

#### 1.1.2. Connexin Hemichannels

While it was originally postulated that connexons were only able to open for communication while docked as GJs, more recent work has suggested that undocked connexons, or hemichannels, do open and close, at least in some situations, to exchange material between a cell and the extracellular space (reviewed in [7,8]). It remains controversial whether hemichannels are active only during pathological states or whether they also open during normal physiological states. Investigating hemichannel function in cultured cells is complicated by the question of whether the effects of hemichannel inhibition are due to beneficial small molecules not able to get into the cell, toxic small molecules not able to get out of the cell, or a combination of the two. Additionally, the study of connexin hemichannel biology is complex due to the presence of pannexin hemichannels. Pannexins form channels that are similar to those composed of connexins, although hexameric pannexin channels in the plasma membrane do not form GJs and instead function as single-membrane channels [9]. It has recently become appreciated that many inhibitors of GJs and connexin hemichannels also inhibit pannexin hemichannels, confounding the interpretation of inhibitor studies in cells that express both connexins and pannexins [10].

#### 1.1.3. Non-Junctional Connexin Functions

In addition to their channel function, connexins are known to mediate extensive protein–protein interactions, which occur primarily through the connexin C-terminal tail. Early work showed a lack of correlation between GJIC and growth suppression [11], suggesting that connexins might have additional functions. Numerous proteins that bind to the intracellular domains of connexins have since been identified, and the importance of some of these interactions in cancer phenotypes is detailed below. Many connexins are also substantially phosphorylated, which alters their ability to interact with other proteins and also affects the gating of the channels they comprise. In fact, channel regulation has been suggested to be the main function of connexin protein–protein interactions, although it is now recognized that there are additional intracellular non-junctional functions as well. However, it can be difficult to separate channel regulation from other connexin activities; ideally, downstream effects of disrupting connexin–protein interactions would be compared to inhibition of GJIC to determine whether the binding affects channel function. Non-junctional functions have also been reported for truncated forms of connexin 43 (Cx43), particularly in the nucleus [12]. Connexins in cancer cells also often become relocalized away from the plasma membrane to intracellular compartments, where they may acquire novel, non-canonical functions. There are many potential intracellular functions of connexins, and this is an emerging area that deserves more in-depth study.

## 2. Mechanistic Roles of Connexins in the Major Cancer Phenotypes

There is an abundance of comprehensive reviews of connexins in cancer [13,14,15,16,17,18,19,20,21,22,23,24,25]. Although the majority of reports on connexins in cancer do not delve into the mechanistic role of connexins, there is a growing number of studies, particularly with the advancement of more specific peptide mimetics and antibodies, that do attribute either GJ activity, hemichannel activity, or a non-channel function of the connexin to the cancer phenotype. Many studies report an association between decreasing connexin expression and increasing tumor grade or show a mislocalization of connexins within the cell and conclude that a loss of GJIC is pro-tumorigenic. However, this could easily be attributable to either connexin hemichannel or non-channel activity, and these alternatives should be tested. Here, we focus on those studies that attempt to isolate which of the connexin functions is responsible for an observed effect on cancer cells, with the aim of adding to our understanding of how connexin expression and function impact cancer phenotypes.

### 2.1. Maintenance of Proliferation and Evasion of Anti-Proliferative Signals

#### 2.1.1. Gap Junction Intercellular Communication (GJIC)

We envision that gap junction intercellular communication (GJIC) could affect cancer cell proliferation through direct effects on proliferative signaling pathways by molecules entering or exiting the cell through the channel—from other tumor cells, from host microenvironmental or immune cells, or through exosomes or tunneling nanotubes, the functions of which have been reviewed elsewhere [26,27,28,29,30,31,32]. Several recent reviews provide a good discussion of what is known about the role of connexins in cancer cell proliferation [13,14,15,17]. Due to the decreased expression of connexin proteins in many cancer cell types, numerous studies hypothesize that GJIC is tumor suppressive, but fewer directly test this hypothesis using specific connexin function modulators rather than adding or removing the proteins. Willebrords et al. provided a comprehensive review of the status of current connexin inhibitors, antibodies, and peptide mimetics [10], some of which have been used in cancer cells. The inhibitor 18-α/β-glycyrrhetinic acid (18-GA), or its derivative carbenoxolone (CBX), while relatively non-specific and non-selective, has been shown to inhibit cell growth in a variety of cancer cell types, including glioma [33], leukemia [34,35], hepatomas [36], and thyroid [37], gastric [38,39], lung [40], bladder [41], prostate [42] and breast cancers [43,44]. Some of these tumors were shown to express connexins (glioma and thyroid), suggesting that the effect might be due to an inhibition of GJIC. However, in other studies, connexin expression was not assessed (leukemia, gastric, breast, lung, and bladder), and the effect of 18-GA may be due to GJIC inhibition or other non-specific effects. Similar to 18-GA, the GJ inhibitor heptanol decreased the proliferation of multiple myeloma cells, which express high levels of Cx43 and demonstrate GJIC, suggesting a pro-proliferative, pro-tumorigenic role for Cx43 in these cells [45]. However, heptanol can also inhibit connexin hemichannels, so this effect cannot be definitively linked to Cx43 or GJIC.

These previous studies suggested that that the degree of GJIC does not correlate with tumorigenicity, which has also been directly observed [46,47], suggesting that the relationship between cell–cell communication and tumor cell proliferation is more complex than simply causative. Subsequently, GJIC has been shown to be pro-proliferative in certain contexts. Treatment of breast cancer cells with the Cx43 targeted peptide mimetic aCT1 increased GJIC and subsequently decreased proliferation [48]. Again supporting a link between alterations in GJIC and proliferation, overexpression of Cx43 decreased proliferation of melanoma cells, and, consistently, treatment with a mimetic peptide that specifically blocks Cx43 GJIC increased proliferation [49]. The use of both inhibitors and peptide mimetics in addition to connexin modulation via genetic approaches increases confidence that the observed effect is truly due to GJIC.

Interestingly, one way that GJs may affect proliferation involves asynchronous cell divisions. Decades ago, it was observed that expression of Cx26 was able to inhibit the proliferation of HeLa cells, while expression of other connexins had little effect [11]. In an elegant mechanistic study, Chandrasekhar et al. showed that this was due to the maintenance of Cx26 GJs at the plasma membrane during cell division, while other connexins are typically internalized during this process [50]. These Cx26 junctions allow the passage of cAMP from cells in M phase into neighboring cells. cAMP then activates protein kinase A (PKA) signaling in G1/S-phase surrounding cells, slowing their cell cycle and suggesting an anti-proliferative role for Cx26. Strikingly, this type of effect may explain how modulation of GJs can affect the growth of homogeneous cultures of cells that should have similar cytoplasmic contents.

Transfer of miRNAs through GJs has also been described to affect cell proliferation. While it is likely that this effect could be pro- or anti-proliferative depending on the miRNA and the cell type, the effects observed thus far have been primarily anti-proliferative. In one example, macrophages coupled with hepatocellular carcinoma cells transferred miR-142 and miR-223 into the cancer cells, inhibiting proliferation [3]. More recently, miR-34a and miR-124-3p were also shown to decrease glioma cell proliferation [4,5], and several miRNAs targeting CXCL12 that were transferred from bone marrow stromal cells slowed proliferation of breast cancer cells [51]. There may be some selectivity to this process, as Cx43 was shown to efficiently transfer miRNAs between cells, with lower levels of transfer for Cx26 and Cx31 and little transfer for Cx32 and Cx37 [5,52]. Thus, the effects of GJIC appear to be both pro- and anti-proliferative depending on the setting.

#### 2.1.2. Connexin Hemichannels

Studies have also described functions for connexin hemichannels in cancer cell growth control, although, as for GJs, specific inhibition of hemichannels remains challenging. However, some studies (e.g., [53]) have used peptide mimetics; pharmacological agents that are relatively specific for hemichannels, such as bisphosphonates; and antibodies specific for the extracellular loops of Cx43, which specifically inhibit hemichannel activity when bound. One critical cargo of connexin hemichannels is ATP. ATP released from hemichannels composed of Cx43 by osteocytes inhibited breast cancer cell proliferation and migration [53], indicating the importance of microenvironmental hemichannel function for tumor cells. However, in other systems, ATP released from pigment epithelial cells through hemichannels increased proliferation of neural retinal progenitor cells [54]. These contradictory effects may in fact be due to the amounts of ATP released into the extracellular space: high concentrations of ATP activate purinergic receptors to induce cell death, while low concentrations may promote cell proliferation [21]. In a non-cancer situation, hemichannel inhibition has also been shown to increase proliferation of rat cardiomyocytes [55] and decrease proliferation of smooth muscle cells [56].

#### 2.1.3. Non-Junctional Roles

As mentioned above, caution should be exercised when assuming that the effects of proteins binding to the intracellular regions of connexins are independent of connexin channel functions. One model of connexin gating proposes that the C-terminal tails of the proteins physically gate the channel, and there is experimental evidence to support this for some connexins [57]. For example, both the channel activity and C-terminal tail of Cx37 are required for rat insulinoma cell proliferation [58,59], but these are not independent; instead, phosphorylation of the C-terminus appears to regulate opening and closing of the GJ, thus regulating proliferation [60,61]. Similarly, phosphorylation of the Cx43 C-terminal tail at S368 by PKA and at Y247 and Y265 by v-src decreases GJIC [62].

However, and perhaps surprisingly, mechanistic studies investigating the non-canonical roles of connexins in cancer cell growth have actually been numerous, likely owing to the relative ease of disrupting protein–protein interactions via genetic means compared to the difficulty involved in studying connexin channels. Many of these functions have been discovered upon observation that the addition of connexin proteins to cancer cells affects cell proliferation without conferring the ability for GJIC, for example, for Cx43 [63] and Cx26 [64] in breast cancer cells. Cx43 inhibits the proliferation of multiple cancer types through channel-independent mechanisms. Expression of only the tail in HeLa cells decreased proliferation [65]; as HeLa cells do not express endogenous Cx43, this suggests a tail-intrinsic effect rather than a dominant-negative effect on GJs or hemichannels. Reports have suggested that Cx43 inhibits basaloid carcinoma cell proliferation through a cytoplasmic role [66], colorectal cancer cell proliferation through an effect on the WNT/β-catenin pathway independent of GJIC [67], glioma cell proliferation by decreasing the activity of SRC through interaction with the Cx43 C-terminal tail [68,69], epidermal growth factor (EGF)-induced ovarian cancer cell proliferation [70], and neuroblastoma cell proliferation [71], among others. Recently, Cx43 was shown to coimmunoprecipitate with both β-catenin and casein kinase, suggesting that Cx43 may exist as part of the “destruction complex” responsible for β-catenin degradation in the absence of WNT ligands [72]. Some of these situations are accompanied by an altered localization of connexin protein, in cases of both overexpressed and endogenous proteins. This has been shown in many cancers, including for Cx43 in glioma [73], rat liver epithelial cells [74], and transformed keratinocytes [75]; Cx26 in breast cancer [64,76]; and Cx32 in liver cancer [77].

Truncated forms of Cx43 have also been shown to affect cell cycle progression. Translation initiating at internal AUG codons within the GJA1 mRNA can produce at least four, and possibly six, different N-terminal truncations of the protein [78,79]. When overexpressed, the 11 kDa form, GJA1-11k, localizes to the nucleus and inhibits transition from G0/G1 phase into S phase of the cell cycle [80]. Other connexins also affect cell cycle progression; for example, Cx37 inhibits the growth of human renal cell carcinoma cells by blocking G1-S phase transition through a channel-independent mechanism involving HER-2 activation [81].

Thus, while the study of connexin functions in the proliferation of cancer cells is difficult due to the frequent decreased expression of connexins, there is evidence that connexins can be both pro- and anti-proliferative. For cells that have lost connexin expression, the ideal type of experiments to study connexin function would be to overexpress a connexin and then use an inhibitor in an attempt to “rescue” the cancer cells, thus attributing the proliferation of the cells to a specific connexin function. The further development of targeting molecules that are connexin and connexin function specific will aid in this process.

### 2.2. Resistance to Cell Death

#### 2.2.1. GJIC

Similar to its role in proliferation, the function of GJs seems to both promote and inhibit cell death. Many studies have linked decreases in GJIC to an induction of cell death, particularly by apoptosis. Treatment of mouse hepatoma cells with chlordane, a benzene metabolite that decreases GJIC [82], enhanced the apoptosis of cells treated with benzo[a]pyrene [83]. In non-transformed cells, treatment of cardiomyocytes with CBX also increased apoptosis [84], and inhibition of GJs via 18-GA and octanol led to apoptosis of endometrial stromal cells [85]. 18-GA and CBX also induced cell death in mouse embryonic stem cells [86]. Furthermore, treatment of thyroid cancer cells with CBX sensitized cells to anoikis, a form of detachment-induced cell death [37]. Knockdown of Cx25 in leukemia cells sensitized cells to cytosine arabinoside chemotherapy [87]. These increases in cell death upon GJ inhibition are perhaps a bit surprising when considering the frequency at which GJ communication is lost in cancer—one might expect that cells that lose expression of connexin proteins would die rather than become transformed. This is consistent with the notion that the ability to evade these apoptotic signals is an essential cancer phenotype.

The presence of functional GJIC is also able to induce cell death, particularly in situations where cancer cells are treated with radiation or chemotherapies. Studies using engineered cells expressing thymidine kinase provided evidence that cells are able to spread pro-apoptotic molecules, in this case likely phosphorylated ganciclovir, through GJs [88,89,90], supporting the idea of a “bystander effect” where cells treated with anti-cancer agents, particularly radiation therapy, are able to induce death in directly adjacent neighboring cells. Subsequent studies have shown that endogenous molecules are also able to spread apoptosis in a GJIC-dependent manner (for example, through Cx43 [91], Cx37/40/43 [92], and Cx32 [93]). The mechanistic basis of how this occurs in vivo is not certain, but some studies have suggested that this effect is potentiated by Ca^2+^/IP_3_ signaling; IP_3_ is required but not sufficient for the activation of apoptosis through GJs [94], and prolonged exposure to high Ca^2+^ levels as induced by IP_3_ in the ER can contribute to cell death [95]. In addition to the bystander effect mediated by radiation, GJIC has been shown to increase chemosensitivity in many models. Use of a dominant-negative Cx43 that blocks GJIC showed that GJIC sensitizes prostate cancer cells to tumor necrosis factor-alpha (TNFα)-induced apoptosis [96]. Cx26 also enhanced hepatocellular carcinoma cell sensitivity to oxaliplatin [97]. Together, these results suggest that GJIC might function to suppress apoptotic stimuli in some cells as it also drives apoptosis in other situations.

#### 2.2.2. Connexin Hemichannels

Similar to GJs, hemichannels can also mediate the bystander effect and spread apoptotic signals between cells. Elegant work in rat C6 glioma cells showed that, while, as one might expect, GJs are able to spread apoptotic signals directly from one cell to another, hemichannel release of pro-apoptotic molecules was able to induce apoptosis in cells up to 100 µm away, suggesting that hemichannels may have a broader scope of an effect on cell death compared to GJs [98]. This effect was negated by Ca^2+^ buffering, suggesting an involvement of calcium ions in this process. It has also been suggested that, due to their effect on intracellular ATP levels, hemichannels may regulate a balance between necrosis and apoptosis [99]; depleted cellular ATP has been shown to drive cells away from apoptosis and toward necrotic cell death [100].

#### 2.2.3. Non-Junctional Roles

In addition to GJ- and hemichannel-dependent roles in apoptosis, channel-independent roles have been described for several connexins. Recently, Cx32 was shown to localize intracellularly in both hepatocellular carcinoma and cervical cancer cells and suppress apoptosis in a channel-independent manner [101,102]. In both cancers, Cx32 bound to SRC, which activates the EGFR signaling pathway, leading to an inhibition of apoptosis induced by the chemotherapy streptonigrin and/or cisplatin. Cx32 also suppressed the induction of apoptosis by TNFα and TNF-related apoptosis-inducing ligand (TRAIL) through activation of the nuclear factor kappa B (NF-κB) pathway in cervical cancer cells [103]. Cx43 has also been shown to induce apoptosis in a channel-independent manner by binding to the apoptotic regulator BAX in both pancreatic cancer and mesothelioma cells [104,105]. In contrast, Cx43 also reduced hydrogen peroxide-induced apoptosis in C6 rat glioma cells by inhibiting caspase activation, perhaps through an interaction with apoptosis signal-regulating kinase 1 (ASK1) [106]. A final example again shows how interconnected connexin functions may be: bisphosphonates inhibit apoptosis of osteoblasts and osteocytes through the activation of extracellular-signal-regulated kinase (ERK) signaling. Plotkin et al. determined that Cx43 hemichannels mediate this signal [107]. The bisphosphonates stimulated Cx43 hemichannels to open, which altered the conformation of the connexins so that SRC could bind, leading to downstream activation of ERK. Based on their frequent loss of expression in cancer cells, one would expect connexins to suppress cancer cell apoptosis, but evidence clearly indicates that they may also be pro-apoptotic in some situations, perhaps dependent on the ability of cancer cells to evade those apoptotic signals.

### 2.3. Replicative Immortality

Only a few studies have reported a mechanistic role for connexins or connexin channels in canonical properties of replicative immortality of cancer cells, such as senescence and immortalization via telomere extension or telomerase expression. More has been reported regarding functions for connexins in the more recently appreciated field of cancer stem cell biology (see below). Several studies have shown changes in connexin gene expression in response to immortalization [108,109,110] but have not investigated the underlying mechanism of connexin function in this situation. A decrease in GJIC with age and with senescence has also been reported [111,112,113,114], as well as changes in connexin expression during senescence [115]. In the context of untransformed cells, knockdown of Cx43 in glomerular mesangial cells increased senescence-associated β-galactosidase staining [116], and Cx43 gene knockout in mesenchymal stem cells also increased cellular senescence. Inhibition of GJIC via 18-GA and octanol decreased telomere length in human endometrial stromal cells [85], suggesting a promoting role for GJIC in immortalization. Nicotinamide adenine dinucleotide (NAD^+^), which can be transferred through GJs and taken up/released by hemichannels, also stabilizes telomeres through its role as a cofactor for sirtuins [117]. Furthermore, specific inhibition of Cx43 hemichannels via the mimetic peptide TAT-Gap19, which inhibits hemichannel activity by binding to the intracellular tail, preventing interaction with the intracellular loop [118,119], decreased radiation-induced senescence [120]. However, an additional study observed no changes in senescence of glioma cells upon overexpression of Cx43 [121]. Together, these studies suggest that connexins and connexin channels may affect the immortality of some cell types, but the direction of this effect may be cell-type specific. This is an area that is primed for future study.

#### Cancer Stem Cells

Cancer stem cells (CSCs, also called cancer- or tumor-initiating cells) are defined as tumor cells that exhibit sustained proliferation; the ability to self-renew or generate an additional stem cell during cell division; and the ability to generate a tumor with similar heterogeneity to the original tumor [122]. Thus, CSCs are important not only for the immortality of a CSC population but also for persistence of a tumor after therapy. The role of GJIC in these cells is complicated not only by the many types and anatomical sites of human cancer but likely also by the variety of CSC populations found within a given cancer. In some instances, it has been observed that CSCs have low expression of connexin genes and exhibit low GJIC (e.g., [123,124,125]; see also reviews on the topic [125,126]). This is supported by some observations in CSCs or CSC-like cells from glioma [127,128], liver cancer [129,130], lung cancer [131,132], triple-negative breast cancer [76], and adenoid cystic carcinoma [133]. However, other studies have shown the presence of GJIC in glioblastoma [33,134], gastric cancer [135], lung cancer [136], and breast cancer [137,138].

Only a few studies have gleaned insight into the mechanisms by which connexin functions affect CSC phenotypes, and, as for other cancer hallmarks, some are contradictory. While studies tend to agree that glioma CSCs express low levels of Cx43, they differ for example on whether and how this is mechanistically important. Cultured glioma CSC-like cells have been shown to express low levels of all connexins tested and to exhibit little GJIC, and re-expression of Cx43 inhibited CSC characteristics including proliferation, self-renewal, and tumor initiation by increasing and binding to E-cadherin, which correspondingly lowered WNT/β-catenin signaling [127]. Other studies have shown similar effects of Cx43 expression in glioma CSCs but attributed its effects to a Cx43-mediated inhibition of SRC kinase activity [139]. This work suggested that Cx43 may suppress the CSC state through channel-independent mechanisms. However, another study observed GJIC in glioblastoma CSCs and found that inhibition of GJIC via CBX or octanol inhibited their growth and self-renewal, as well as tumor growth in vivo [33]. These CSCs expressed lower levels of Cx43 compared to non-stem cells, as previously reported [127], but relatively higher levels of Cx46 [33]. Blockage of GJIC by the seemingly Cx46-specific phenazine dye clofazimine or expression of a Cx46 GJIC-incompetent mutant phenocopied the use of more general GJ inhibitors and suggested that glioblastoma CSCs may rely on Cx46-mediated GJIC [134].

While, to our knowledge, no role for connexin hemichannels in CSC biology has yet been reported, several other interesting non-membrane functions have been described. Cx26 is expressed at relatively higher levels in triple-negative breast cancer CSCs compared to non-stem cells and maintains the properties of these cells via an intracellular complex with focal adhesion kinase (FAK) and the pluripotency transcription factor NANOG [76]. Intracellular Cx32 expression in hepatoma cells also increased the self-renewal of cells, suggesting that Cx32 may play a cytoplasmic role in maintaining a CSC-like state [140]. Thus, it is likely that connexin proteins exert both CSC-promoting and CSC-inhibitory effects depending on the connexin and the biological system, and, as the field of CSC biology continues to mature, further study may shed additional light on this phenomenon and the mechanisms underlying it.

### 2.4. Angiogenesis

The growth of new blood vessels within a tumor is critical for the delivery of oxygen and other nutrients. Knockdown of any of the major connexins expressed in endothelial cells, Cx43, Cx37, and Cx40, compromises endothelial branching in vitro [141], and Cx43, Cx37, and Cx40 knockout mice also exhibit vasculogenic/angiogenic defects [142,143,144]. While these studies show the importance of connexins for vessel growth and remodeling, they do not provide mechanistic insights into the functions of connexins that are necessary for these processes. Although coupling of tumor cells with endothelial cells is also important for cancer cell migration and extravasation (discussed next), here, we focus specifically on angiogenic processes.

#### 2.4.1. GJIC

Communication between tumor cells and endothelial cells in the microenvironment is necessary for stimulation of angiogenesis. In glioma, Cx43 has been suggested to be both pro-angiogenic and anti-angiogenic. On the pro-angiogenic side, co-culture of glioma cells expressing Cx43 with human umbilical vein endothelial cells (HUVECs) stimulated HUVEC tube formation, an angiogenic process [145]. Functional GJs formed between the two cell types, and, because the effect on tube formation required direct cell–cell contact, it is likely that this effect was due to GJIC. Similarly, in a non-cancer setting, bone marrow mononuclear cells activate angiogenesis by GJIC with endothelial cells [146]. However, anti-angiogenic roles have also been reported. Knockdown of Cx43 in human glioma cells increased their ability to induce angiogenesis [147], although the mechanism of this effect was not clear. Similarly, breast cancer cells release the proliferative inhibition of endothelial cells by mural cells by secreting a signal that inhibits Cx43-mediated GJIC between these two cell types [148]. miRNAs transported through GJs have also been shown to affect angiogenesis. GJ-mediated transfer of miR-145-5p from human microvascular endothelial cells to colorectal cancer or glioblastoma cells inhibits the ability of the cancer cells to stimulate angiogenesis [149,150]. This is accompanied by a reciprocal transfer of miR-5096 from tumor cells to endothelial cells, which stimulates tubulogenesis of HMECs and therefore appears to be pro-angiogenic [150]. Thus, interestingly, GJIC may be both pro- and anti-angiogenic even in the same cancer. Finally, Cx40 has been shown to have a pro-angiogenic function in tumor cells. Inhibition of Cx40-mediated GJIC in a mouse lung tumor model via treatment with ^40^Gap27, a GJIC-specific peptide mimetic inhibitor of Cx40, reduced tumor growth and angiogenesis [143]. These results suggest then that GJIC, particularly that mediated by Cx43, has a range of effects on endothelial cell proliferation and tumor angiogenesis.

#### 2.4.2. Connexin Hemichannels

While, to our knowledge, no documented effects of connexin hemichannel function on tumor angiogenesis have been reported, it is tempting to speculate that many molecules found in the tumor microenvironment that would be permeable to hemichannels may affect vessel formation. Nucleotides including ATP and UTP released from breast cancer cells stimulated P2Y purinoceptor 2 (P2Y2R) [151], which can induce vascular sprouting [152]. Ca^2+^ [153,154] and the cAMP-dependent kinase PKA [155] have also been shown to affect angiogenesis. Overexpression of Cx43 in mouse melanoma and breast cancer cells inhibited angiogenesis, and this was mediated by a soluble factor in conditioned medium, suggesting that this could be due to hemichannel release of some molecule [156,157,158]. Thus, while no clear roles for hemichannel activity of connexins have been documented at this point, it is possible and even likely that hemichannels present on tumor cells or other microenvironmental cells may play a role in tumor angiogenesis.

#### 2.4.3. Non-Junctional Roles

Similar to hemichannel involvement in angiogenesis, no clear roles for non-junctional functions of connexins have been described in angiogenesis, but these effects likely occur. Comparison of a GJIC-incompetent mutant of Cx26 with wild-type Cx26 suggested that Cx26 controls the expression of angiogenesis-associated genes through both GJ-dependent and GJ-independent mechanisms [159], as expression of both forms of the protein upregulated expression of thrombospondin, an anti-angiogenic protein. This GFP-Cx26 appears to be non-functional for GJIC or as a hemichannel, suggesting that this is a non-junctional role [159]. Together, it is clear that GJIC is the best understood role of connexins in tumor angiogenesis, but there is potential for the discovery of additional roles.

### 2.5. Invasion and Metastasis

Other than functions in cancer cell proliferation, the role of connexins in migration, invasion, and metastasis is the most frequently studied and has been comprehensively reviewed (e.g., [13,17,160,161,162,163]). Here, again, we focus on developing a mechanistic understanding of how connexin functions promote or inhibit invasion and metastasis.

#### 2.5.1. GJIC

The role of GJs during invasion and metastasis likely relates back to the role of connexins during different stages of tumorigenesis (diagrammed nicely in [14,20]). It has been proposed that connexins more frequently function to suppress the early stages of transformation (although, again, this is cancer and connexin dependent) but enable later stages such as metastasis. Particularly in the context of invasion and metastasis, GJs may serve an adhesive function in addition to their communicative role (reviewed in [6,164,165]). One could envision a situation where, during metastasis, a decrease in any homocellular GJs present between cancer cells would first need to occur, followed by an increase in heterocellular GJs between cancer cells and microenvironmental cells, both to enable migration and to establish a metastatic lesion in a satellite location.

One of the most striking examples of a role for heterocellular GJIC during tumor metastasis was provided by the Massagué group for breast cancer metastasis to the brain. This model suggests that metastatic breast cancer cells form functional heterocellular GJs composed of Cx43 with astrocytes, and transfer of cyclic guanosine monophosphate-adenosine monophosphate (cGAMP) from tumor cells to astrocytes through these GJs stimulates the production of inflammatory cytokines by the astrocytes [166]. These cytokines then reciprocally activate the signal transducer and activator of transcription 1 (STAT1) and NF-κB pathways in the tumor cells, ensuring the proliferation of the metastatic cells. Similarly, work in glioma suggested that homocellular GJs between glioma cells inhibit metastasis, while heterocellular GJs formed between glioma cells and astrocytes promote metastasis in a GJIC-dependent manner [167]. However, this contrasted a study by the Naus laboratory indicating that, while functional GJIC between astrocytes is required for glioma invasion, GJIC between astrocytes and glioma cells is dispensable [168]. Furthermore, both homocellular GJIC between breast cancer cells and heterocellular GJIC between breast cancer cells and endothelial cells promoted cancer cell diapedesis and thus invasion and metastasis [169]. Other studies also support the idea that GJIC promotes tumor metastasis (in breast cancer [170] and lymphoma [171], Cx43 and Cx26 in breast cancer and melanoma [172], Cx26 in melanoma [173,174], Cx43 in breast cancer [175], Cx43 in glioma [168,176,177], Cx43 in non-small cell lung cancer [178], and Cx43 in prostate cancer [179], among other studies). However, suppression of metastasis by GJIC has also been reported, often even for the same connexins in the same types of cancer (Cx26 and Cx43 in breast cancer [157], Cx43 in glioblastoma [180], Cx43 in breast cancer [181], and Cx32 in cervical cancer [182]). A recent study used a GJIC-incompetent Cx43 mutant to show that Cx43 GJIC is required for suppression of epithelial-to-mesenchymal transition (EMT) genes by osteosarcoma cells when cultured with osteoblasts, suggesting that heterocellular GJIC between these two cells types restrains metastatic potential [183]. Thus, additional work is required to resolve these conflicting results and clarify the role of GJIC in tumor invasion and metastasis, ideally through the use of an in vivo model system to ensure the presence of a tumor microenvironment. In vivo studies of cancer cell migration and invasion would particularly benefit from the development of new tools to target GJIC, including inhibitors with increased specificity or approaches to engineer tissue-specific expression of connexin mutants.

#### 2.5.2. Connexin Hemichannels

While it may be difficult at times to experimentally distinguish between communication and adhesion mediated by GJs in metastasis and invasion, reported roles for connexin hemichannels would suggest that cells can receive pro- and/or anti-metastatic signals from the microenvironment in addition to signals from other cells. Several studies have linked connexin hemichannels and migration of non-transformed cells. In the context of the normal brain, Cx43 hemichannels are necessary—but not sufficient—for neuron-induced astrocyte migration [184,185], and calcium waves released through hemichannels have been proposed to play a role in neuronal proliferation and migration during development [186]. There are only a few studies that document a clear role for connexin hemichannels during invasion and metastasis. The first showed a microenvironmental role for hemichannels rather than a tumor cell-intrinsic role: open hemichannels on osteocytes create high ATP concentrations in the extracellular space, which inhibits the metastasis of breast cancer cells to the bone [53]. This leads to the question of whether hemichannels are then active on osteocytes in a non-tumor context or whether the presence of tumor cells modifies the behavior of microenvironmental bone cells, which would have implications for the assumption that hemichannels open primarily in disease states. Subsequent studies have also shown pro-metastatic roles for hemichannels on cancer cells. Recent work showed that breast cancer cell collective invasion occurred via hemichannel-released adenosine nucleosides/nucleotides, which activated purinergic receptors in an autocrine fashion to promote leader cell function [187]. Finally, a fascinating report suggested that, in addition to the passage of small molecules, hemichannels can also function for adhesion. The expression of high levels of Cx43 in glioma cells stimulated adhesion to cells with low Cx43 expression [188]. While this study did not rule out alterations in other cell adhesion markers or mechanisms in response to Cx43 expression, the idea that connexin hemichannels may play a role in cell adhesion may deserve additional study in metastasis.

#### 2.5.3. Non-Junctional Roles

Compared to other cancer phenotypes, there is comparatively more information available regarding a non-junctional role for connexins in cancer cell invasion and metastasis, with both pro- and anti-metastatic functions described depending on context. In glioma cells, the C-terminal tail of Cx43 was sufficient to stimulate motility in a GJIC-independent manner (although the channel domain alone also stimulated migration, suggesting that Cx43 may induce glioma cell invasion through multiple mechanisms [189,190]). This tail-mediated mechanism might involve cytoskeletal rearrangement driven by interaction with cellular communication network factor 3 (CCN3), an integrin ligand involved in adhesion, as was shown for breast cancer cells [191]. Non-junctional pro-metastatic roles for Cx43 have also been described in prostate cancer [179,192], ovarian cancer [193], and cervical cancer [194], with additional non-junctional functions reported for Cx32 in hepatoma cells [195]. Studies on non-junctional, anti-metastatic connexin functions have been pioneered by the Laird laboratory, particularly for breast cancer. Cx26 inhibited breast cancer cell invasion through Matrigel by reducing matrix metalloproteinase (MMP) activity and integrin β1 levels even when localized intracellularly [64]. Similarly, Cx43 suppressed the invasion of transformed keratinocytes in a GJIC-independent manner via C-terminal interaction with caveolin-1 [196]. Suppression of invasion has also been shown for Cx32 in renal cell carcinoma cells, via a SRC-STAT3-vascular endothelial growth factor (VEGF) pathway [197], and for Cx43 in prostate cancer cells when localized intracellularly [179]. Translocation of the truncated GJA1-20k to the nucleus also stimulates N-cadherin expression and drives collective cell migration [198]. As for GJIC, there is still much work to do to understand non-junctional roles for connexins in tumor metastasis and invasion, but it is apparent that connexins play a substantial role in these processes.

## 3. Mechanistic Roles of Connexins in More Recently Appreciated Cancer Phenotypes

In their updated “Hallmarks of Cancer: The Next Generation,” Hanahan and Weinberg added what they termed “emerging” or “enabling” characteristics of cancers [2]. Connexins play a role in each of these four cancer phenotypes, which include altered energy metabolism, avoiding the immune system and inducing inflammation, and genetic instability, and, to this point, the majority of functions that have been described appear to be channel dependent (with the exception of a few examples for genomic instability, as noted below). Particularly for metabolism, it is easy to see how the transfer of small metabolites through GJs is likely to play a role in cancer. The potential for non-junctional roles of connexins in these cancer phenotypes represents an opportunity for further discovery.

### 3.1. Alterations in Energy Metabolism

#### 3.1.1. GJIC

Cancer cells require high amounts of energy to support increased proliferation, and they typically take up high amounts of glucose to provide this energy. However, rather than utilizing oxidative phosphorylation to produce ATP from this glucose, cancer cells often divert their glucose metabolism to an aerobic glycolytic pathway, which produces lower amounts of ATP more quickly and also less reactive oxygen species (ROS), along with more lactate [199]. As described above, molecules such as glucose, as well as ATP, AMP, guanosine triphosphate (GTP), cAMP, and other small energy carriers, are able to pass through GJs. In fact, in glioma cells, ATP and ADP made up more than 6% of the total material derived from labeled glucose that passed through GJs [200], and the passage of ATP through GJs has been implicated in many of the abovementioned cancer phenotypes.

For this reason, it is clear that altered expression of connexins or altered function of GJIC or hemichannels on cancer cells is likely to affect the energy resources of both tumor cells and the microenvironment. This has been well described in the context of normal astrocytes and transformation into malignant astrocytomas such as gliomas [201]. The loss of Cx43 in normal astrocytes, which presumably decreases glucose influx and thus the total amount of glucose available to the cell, drives an increase in membrane localization of the GLUT-1 and GLUT-3 glucose transporters [202], increasing glucose uptake [203]. This is a tempting model for alterations in glucose import in response to transformation of normal astrocytes into astrocytomas/gliomas driven by a loss of Cx43, particularly as gliomas primarily obtain glucose through high expression of GLUT1/3 [204]. However, the situation does not seem to be so simple, as restoring Cx43 function to rat C6 glioma cells did not alter GLUT1/3 localization or expression [205]. Instead, increased GJIC caused a release of hexokinase from the mitochondrial membrane, which results in decreased enzymatic activity and lowered ability to phosphorylate glucose, the first step of glycolysis [205]. Thus, an absence of GJIC is likely to play an important role in the ability of cancer cells to maintain glycolysis, although the mechanism of how GJIC alters hexokinase localization remains to be determined.

Tumors are heterogeneous and contain perivascular (oxygen- and nutrient-rich) areas and hypoxic (oxygen- and nutrient-poor) areas, in addition to a variety of cancer and non-transformed cell types. In conditions of hypoxia, cancer cells primarily produce energy through glycolysis, which leads to a buildup of acidic byproducts [206]. Coupling between normoxic and hypoxic tumor cells via GJs allows the passage of HCO_3_^−^ ions from normoxic to hypoxic cells, which helps neutralize H^+^ ions [206]. In this way, Cx43 GJs may help maintain hypoxic tumor cells. Cancer-associated fibroblasts (CAFs), which are found in the tumor microenvironment, form GJs with non-small cell lung cancer (NSCLC) cells, which drives aerobic glycolysis in the CAFs and increased oxidative phosphorylation in the cancer cells [178]. This leads to increased activation of the phosphatidylinositol 3-kinase (PI3K)/AKT and mitogen-activated protein kinase (MAPK)/ERK pathways and cancer cell invasion by inducing EMT. Together, these studies all support a model where Cx43-mediated GJIC affects tumor cell metabolism, although some studies show that GJIC promotes tumor properties and some show that GJIC suppresses tumor properties. This again may be context dependent, perhaps a situation where heterocellular GJIC aids in tumor growth, while homocellular GJIC between tumor cells suppresses it.

#### 3.1.2. Connexin Hemichannels

Connexin hemichannels have been shown to release NAD^+^, a co-enzyme involved in many metabolic pathways [21,207], suggesting that hemichannels may work to support high levels of glycolysis in surrounding cells while potentially decreasing glycolysis in cancer cells themselves. Additionally, while it remains to be seen whether this is also the case in cancer cells, Cx43 has been shown to localize to mitochondria, in particular the inner mitochondrial membrane [208,209], in cardiomyocytes. It appears likely that the protein exists as part of a functional hemichannel structure there, as cross-linking studies suggest the presence of connexin hexamers that are capable of transfer of Lucifer yellow and can be inhibited by CBX and heptanol [208]. Treatment with 18-GA showed that these hemichannels appear to be important for mitochondrial K^+^ flux, the inhibition of which is important for the maintenance of cancer cells, with restoration of normal flux leading to apoptosis [210]. K^+^ flux is also involved in mitochondrial respiration [211,212], and, consistently, inhibition of mitochondrial hemichannel Cx43 reduces mitochondrial ATP production via respiration [213]. Furthermore, Cx43 has also been shown to regulate Ca^2+^ entry into the mitochondria in the heart, potentially leading to cell death [214]. Thus, connexin hemichannels release and take up molecules that play roles in metabolic pathways, and, although connexin localization in the mitochondrial membrane has not been shown in cancer, this represents an intriguing possibility to directly affect the metabolic state of cancer cells.

### 3.2. Inflammation and Immune Evasion

#### 3.2.1. GJIC

Over the past two decades, an appreciation has developed for the role of GJs in immune function and inflammation in cancer, with primarily anti-tumorigenic roles reported (reviewed in [215,216,217]). Connexins are expressed on most types of immune cells, and GJIC is important for a variety of immune cell functions, including hematopoiesis, antigen presentation, phagocytosis, migration, and inflammation, among others [215]. Combination treatment with the pro-inflammatory cytokines TNFα and interferon gamma (IFNγ) upregulates Cx43 expression on monocytes/macrophages [218] and microglia [219] and leads to a transient homocellular GJ coupling. Inhibition of this newly established GJIC via 18-GA decreases transmigration of monocytes across a model blood–brain barrier, suggesting that immune cell GJIC stimulated by inflammation may play a role in the recruitment of immune cells to tumor sites (a potential anti-tumor role).

GJIC also plays a role upstream of TNFα and IFNγ production: the transfer of the second messenger cGAMP from tumor cells to astrocytes, macrophages, and dendritic cells (DCs) via GJIC induces expression of pro-inflammatory cytokines [166,220,221] and leads to activation of CD8+ T cells that target cancer cells [220]. However, cGAMP transfer can also be pro-tumorigenic by leading to the proliferation of tumor cells [166], indicating that inflammation serves multiple roles in the context of cancer. GJIC is also involved in antigen presentation through direct coupling between monocytes and DCs [222], between DCs [223], between tumor cells and endothelial cells [224], and at the immune synapses formed between melanoma cells and CD4+ T cells [225], melanoma cells and natural killer (NK) cells [226], and DCs and CD4+ T cells [227]. Moreover, GJIC between tumor cells and NK cells inhibited tumor cell targeting by NK cells [226]. Coupling between macrophages and tumor cells has also been shown to transfer tumor-suppressive miRNAs to the cancer cells [3]. These examples suggest that the ability of the immune system to target cancer cells is increased by: (1) heterocellular GJIC between tumor cells and immune cells; (2) heterocellular GJIC between different types of immune cells; and (3) homocellular GJIC within a population of immune cells, further supporting an anti-tumorigenic role for connexins, with variable effects of pro-inflammatory cytokines. As immunotherapies are further tested and developed as anti-cancer therapies, the role of GJs and the potential of treatments to increase GJIC should be considered.

#### 3.2.2. Connexin Hemichannels

It is clear that, in addition to GJs, hemichannels also localize to the immune synapse [228]. However, their function there is less understood. The release of ATP by hemichannels on activates purinergic receptors on T cells in an autocrine manner, which is necessary for T cell activation [229]. However, while connexin hemichannels do release ATP [230], pannexins appear to be more important for ATP-mediated activation at the immune synapse, as preferential inhibition of pannexin channels had a much greater effect on T cell activation compared to connexin hemichannel inhibition [229,231]. It has also been reported that Cx43 hemichannels on T cells are important for T cell proliferation [232]. A role for pannexins was not excluded here, but the use of a blocking antibody (Gap7M) that binds to the extracellular domain of several connexins would suggest specificity for connexin hemichannels. Thus, while connexin hemichannels have the potential to drive T cell activation, in practice, it seems that they may have a more established role in T cell proliferation. Furthermore, release of NAD^+^ through hemichannels also has the potential to drive an anti-tumor immune response (reviewed in [21]). NAD^+^ has a variety of roles in immune cells (reviewed in [233]), including determining T cell fate.

However, there has also been a documented role for connexin hemichannels in promoting inflammation (reviewed in [234]), potentially through the release of ATP to activate cytokine release by macrophages. Connexin mimetic peptides that block hemichannel function, including aCT1 and JM2, decrease inflammation during wound healing [235,236] and in response to CNS injuries [237,238]. When these observations are taken together, connexin hemichannels seem to have divergent functions, as they are important for the function of the immune system to activate an anti-tumor response but also drive inflammation that promotes tumor growth.

### 3.3. Genomic Instability

#### 3.3.1. GJIC

Connexins impact genomic stability in a variety of ways, some direct and some indirect. Inflammation, as detailed above, can lead to DNA damage and genomic instability, suggesting that connexin functions that promote inflammation might then affect DNA stability [239]. GJIC plays a major role in the bystander effect, whereby irradiated cells can spread DNA damage-causing molecules to non-irradiated cells via GJIC [240], likely through the generation of reactive oxygen species (ROS) in the irradiated cell and, through the bystander effect, surrounding cells (reviewed in [241]). This phenomenon has been termed the “kiss of death,” whereby cells share their toxic signals, including ROS molecules themselves [242], with surrounding cells [243]. In addition to irradiation, ROS can be generated through multiple mechanisms in which GJIC plays a role, including tumor cell metabolism and inflammation, and, through this role in DNA damage, can be pro-transformative (reviewed in [244]). However, GJIC can also provide a “kiss of life” [243], where the presence of functional GJs protects cells against oxidative stress and thus genomic instability by allowing the outward diffusion of pro-death signals; for example, GJIC protected human retinal pigment epithelial cells from death induced by a chemical oxidant [245] and astrocytes from ROS-induced oxidative stress [246]. The situation becomes even more complex when considering that antioxidants such as glutathione can also pass through GJs [247], suggesting that the local cellular concentrations of ROS and antioxidants will determine whether GJIC promotes or inhibits ROS-mediated DNA damage. Similarly, the degree to which ROS damage DNA and the ability of a cell to repair this damage will affect whether the DNA damage promotes transformation or is lethal to the cell.

#### 3.3.2. Connexin Hemichannels

Most of the roles of GJIC in genomic instability also apply to hemichannels. Even the bystander effect, which is often thought of as requiring a direct coupling between cells, can be mediated by soluble factors in conditioned media, potentially through release and uptake by hemichannels [248]. This effect was recently shown to be inhibited by the hemichannel function-specific Cx43 mimetic peptide Gap19, suggesting that this effect is in fact due to hemichannels [120,249]. Both DNA damage-inducing ROS [120,246,249] and the antioxidant glutathione [250,251] are also able to pass through hemichannels. NAD^+^, which can be released and taken up by hemichannels [252], also plays a role in genomic stability through the activity of sirtuin deacetylases [253,254]. The enzymatic activity of sirtuins requires NAD^+^ [255], and SIRT2 and SIRT6 confer resistance to DNA damage by deactylating histones and inhibiting DNA recombination [253,254]. Cx43 has been proposed to act as a histone deacetylase inhibitor in a manner involving soluble factors [256], and this indirect effect on sirtuins mediated by NAD^+^ release may be the underlying mechanism for this observation. Thus, as for GJs, the effects of connexin hemichannel activity on DNA damage and genomic instability likely depend on context, with the intra- and extracellular concentrations of molecules determining whether they are pro- or anti-tumorigenic.

#### 3.3.3. Non-Junctional Roles

One interesting study showed a non-channel role for Cx43 in genomic instability. Transfection of HeLa cells with Cx43 cDNA decreased genomic instability, but this effect was not lost upon treatment with 18-GA [257]. This suggests a fundamental role for Cx43 in maintaining genomic stability that is not mediated by the transfer of small molecules but rather by the protein itself, although the mechanisms of this remain to be determined. Less directly, expression of the GJA1-20k truncation form of Cx43, which contains part of the fourth transmembrane domain and the C-terminal tail (see [12]), in the heart decreased cellular ROS [258]. However, GJA1-20k acts as a type of chaperone to recruit full-length Cx43 to the plasma membrane, suggesting that it may also impact its channel activity [79]. Thus, it is not clear whether this effect is due to a channel-dependent or a channel-independent function of the protein. Regardless, the potential for non-channel functions of connexins to maintain genomic stability is fascinating and deserves further study.

## 4. Conclusions and Future Perspectives

**Figure 1:**
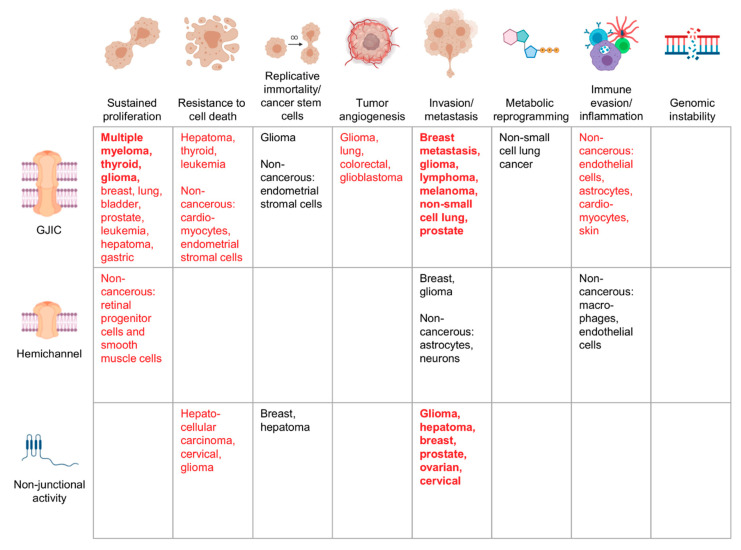
Cancers in which specific connexin functions have been described to be pro-tumorigenic. Schematic showing examples of the cancer types in which pro-tumorigenic properties have been attributed to a specific connexin function, whether gap junction intercellular communication (GJIC), connexin hemichannels, or a non-junctional function. Bold red text indicates strong evidence from many cancer types tying the connexin function to the corresponding cancer hallmark, with red text indicating a less robust link and black text indicating emerging evidence. Where mechanistic studies in cancer cells are not available, certain non-transformed cell populations are indicated. References for the indicated cancers are provided in the text. Figure illustrations generated using BioRender.com.

**Figure 2:**
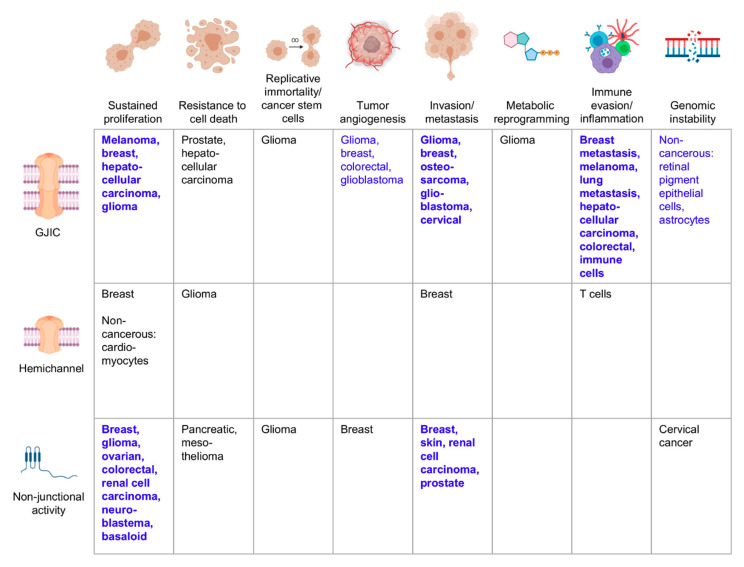
Cancers in which specific connexin functions have been described to be tumor suppressive. Schematic showing examples of the cancer types in which tumor suppressive properties have been attributed to a specific connexin function, whether gap junction intercellular communication (GJIC), connexin hemichannels, or a non-junctional function. Bold blue text indicates strong evidence from many cancer types tying the connexin function to the corresponding cancer hallmark, with blue text indicating a less robust link and black text indicating emerging evidence. Where mechanistic studies in cancer cells are not available, certain non-transformed cell populations are indicated. References for the indicated cancers are provided in the text. Figure illustrations generated using BioRender.com.

Over the past 60 years, the field has advanced from the observation that cancer cells often lose communication and metabolic coupling to an understanding of many of the molecular and functional mechanisms underlying the role of connexins in cancer (Figure 1 and Figure 2). As our knowledge continues to expand, it becomes clear that, despite the variety of connexin proteins and functions, many of the roles executed by connexins in cancer cells are tightly interconnected. The transfer and uptake/release of small molecules including ATP and NAD^+^ are upstream of most—if not all—cancer phenotypes, supporting the hypothesis that altered connexin activity may be a unifying hallmark of cancer [259]. Remaining questions include the identity of essential molecules being transferred, the functional effects of connexin mislocalization, and the differential selectivity of different combinations of connexins. There is also a lot still to be learned about non-junctional roles for connexin proteins in cancer.

When evaluating connexin function in cancer, it is important to note that the role of a connexin in a given cancer cell will depend on the context, including the cell and tumor type, the connexin isoforms expressed in the cell, and the specific microenvironment of the cell (including the concentration of extracellular molecules and the surrounding cells available for GJ coupling), as well as the mutational and expression profiles of the cell. While mutations in connexin genes typically occur with only a low frequency in cancers [14], these alterations in connexin sequence likely affect function in some cases. The importance of GJIC between cancer cells (homocellular communication) may be very different from the necessity of communication between a tumor cell and a non-transformed cell in the microenvironment (heterocellular communication). Differences in context likely underlie the many contradicting reports of connexin functions in cancer cells. Furthermore, relevant to the discussion presented here, multiple cancer phenotypes likely lie downstream of one connexin function, for example, the multiple roles of ATP, NAD^+^, and ROS transfer through GJs and uptake and release by hemichannels.

It has been challenging to determine whether alterations in connexin expression observed in tumor tissues have a direct or indirect effect on cancer cell phenotypes. The use of knockdown/knockout approaches has facilitated an understanding of these effects in animal models and cultured cells, but these techniques still do not shed light on the mechanisms underlying the effect of alterations in connexin expression. Furthermore, cells may compensate for removal of one connexin by upregulating another family member, further complicating the situation. One challenge of the field is the small number of truly specific inhibitors that target a given function of a given connexin. The development of better inhibitors is complicated by the high degree of similarly among connexins and the complex interplay among connexin functions. Non-specific inhibitors of GJs, including CBX, oleamide, mefloquine, octanol, 18-GA, and others [260], have been used and have contributed valuable knowledge to the field. However, these types of inhibitors are not ideal due to their off-target or non-specific effects and their lack of selectivity for specific connexin proteins and specific connexin functions [260]. Work on Cx43 has been dramatically aided by the development of the Cx43 mimetic peptides that target one or more connexin function, although these peptides also modulate the functions of other connexins to varying degrees [10]. While the most mechanistic work has been performed for Cx43, it is clear that other connexins also have roles in cancer phenotypes. For other connexins, the only options to isolate the role of GJ vs. hemichannel vs. non-channel functions remain either nonspecific inhibitors or mutation of the connexin. Connexin point mutations bring about their own set of challenges, including requiring either altering the levels of protein expressed via overexpression or genome editing, as well as a detailed understanding of the structure–function relationships within the molecule. However, connexin point mutations may still affect more than one connexin function concurrently (for example, GJ and hemichannel activity or protein–protein binding and channel function), confounding the interpretation of results.

Although the existence of connexin hemichannels has become more accepted in the field, their study continues to be complicated by the presence of pannexin hemichannels, which are sensitive to many of the same inhibitors as connexin channels [10]. The development of approaches to target the functions of connexins other than Cx43 without disrupting pannexin function will greatly aid our understanding of connexins in cancer and provides an immense opportunity to gain insight into the importance of connexin activity in both cancer and normal physiology. These types of inhibitors will also have a high therapeutic potential to treat the multitude of pathological conditions, including cancer, in which connexins are involved.
